# Extraction and Characterization of Inulin-Type Fructans from Artichoke Wastes and Their Effect on the Growth of Intestinal Bacteria Associated with Health

**DOI:** 10.1155/2019/1083952

**Published:** 2019-09-25

**Authors:** Zahraa Zeaiter, Maria Elena Regonesi, Sofia Cavini, Massimo Labra, Guido Sello, Patrizia Di Gennaro

**Affiliations:** ^1^Department of Biotechnology and Biosciences, University of Milano-Bicocca, Piazza della Scienza 2, 20126 Milan, Italy; ^2^CRC Materiali Polimerici (LaMPo), Department of Chemistry, University of Milan, via Golgi 19, 20133 Milan, Italy; ^3^Department of Chemistry, University of Milan, via Golgi 19, 20133 Milan, Italy

## Abstract

Globe artichoke is an intriguing source of indigestible sugar polymers such as inulin-type fructans. In this study, the effect of ultrasound in combination with ethanol precipitation to enhance the extraction of long chain fructans from artichoke wastes has been evaluated. The inulin-type fructans content both from bracts and stems was measured using an enzymatic fructanase-based assay, while its average degree of polymerization (DP) was determined by HPLC-RID analysis. Results show that this method provides artichoke extracts with an inulin-type fructans content of 70% with an average DP between 32 and 42 both in bracts and in stems. The prebiotic effect of long chain inulins from artichoke extract wastes was demonstrated by its ability to support the growth of five *Lactobacillus* and four *Bifidobacterium* species, previously characterized as probiotics. Besides, we considered the possibility to industrialize the process developing a simpler method for the production of inulin-type fructans from the artichoke wastes so that the artichoke inulin preparation could be suitable for its use in synbiotic formulations in combination with different probiotics for further studies including *in vivo* trials.

## 1. Introduction

The human intestine is home to approximately 10^14^ functional microorganisms including bacteria, fungi, yeasts, viruses, and protozoa [1]. The stability and diversity of this population is hindered by stress, infection, antibiotic use, and other environmental factors resulting in gut dysbiosis in some cases [2], that can be associated with many health issues and infections.

In this regard, the consumption of live microbial feed supplements defined as probiotics or beneficial bacteria are thought to equilibrate this population and to prevent and treat a variety of diseases, among them: reduction of lactose intolerance symptoms, decrease of cholesterol levels, irritable bowel syndrome (IBS), protection against pathogenic bacteria, and reestablishment of intestinal flora after antibiotic therapy [3, 4]. The majority of microorganisms commonly employed as probiotics belong to the genera *Lactobacillus* and *Bifidobacterium *[5]. In human body, they should adhere to the intestinal cells, proliferate and colonize the intestine to limit the activity of pathogen microorganisms, and allow the interaction with the immune cells [6]. Therefore, the beneficial physiological effects attributed to probiotic bacteria are dependent on the strain and the number of viable bacteria that reach the gastrointestinal system which make their viability a prerequisite for their functionality.

On the other hand, prebiotics are dietary carbohydrates that bypass the hydrolysis in the upper part of the gastrointestinal system and are selectively used by host microorganisms, conferring health benefit to the host [7]. Prebiotics are employed in synbiotic preparation along with probiotic strains to favor the viability of these bacteria in the colon as reported in the recent review by Krumbeck et al. [8]. Inulin-type fructans are the most commonly studied and used prebiotics [9]. They consist of repetitive chain of fructosyl moiety, linked by *β*(2,1) bonds with terminating glucosyl moieties. Inulin molecules are found in a variety of vegetables such as asparagus, leeks, artichokes, onions, and garlic items with varying degree of polymerization (DP) which is associated to their different functional features [10]. The extracted inulin from these plants are known for their therapeutic, preventive, and physioprotective effects as lowering of blood cholesterol or glucose level by reducing lipogenesis and the antioxidant effects [9–13]. Moreover, these inulin-type fructans selectively stimulate also the growth of *Bifidobacterium* and *Lactobacillus* spp. in the human colon and as a result of their fermentation, short chain fatty acids (SCFA) can be formed. The latters lower colon pH and subsequently enhance the absorption of mineral ions (Ca^2+^ and Mg^2+^) and nutrients in the host body [14].

Regarding the health beneficial potential of inulin, its extraction from natural source has become a subject of interest in many food research programs [9]. Recently, with the sustainable technological development, the exploitation of waste biomass generated by plant or food processing in the production of high added-value functional ingredients to be applied in the nutraceutical and pharmaceutical industry has become a focus. In particular, artichoke plant represents an intriguing source of unique functional molecules like dietary fibers and bioactive compounds [15–19]. As well, Italy is the foremost producer country of artichokes, accounted to 33% of global production. Accordingly, the organic wastes generated by artichoke cultivation are an abundant natural biomass source, where the stems remain mostly in the ground and are not harvested or consumed. Besides, artichoke canning industry employs a small part of the artichoke plant in canning food while it generates a large amount of industrial waste consisting mainly of external parts of the artichoke flowers (bracts) which are not suitable for human consumption and could be only used as livestock food. These parts of the plant considered as waste and which compose mainly the 70% of the whole plant weight, can be recycled for the production of compounds of commercial interest [20, 21], such as inulin to supply as prebiotic for probiotic strains. Moreover, the majority of strains were reported to degrade only short chain fructans (fructooligosaccharide DP <10) (FOS), while limited number of probiotics has the capacity to metabolize long chain inulin-type fructans [22, 23]. Strains with this ability are very important, because long chain inulins affect the production of short chain fatty acids when they are used as carbon source for growth; e.g., butyrate, known for its antitumor and other beneficial effects on human health, is mainly produced by inulin fermentation [22]. Recently, it was also demonstrated that long chain inulins of DP 10–60 exert higher immune response in human than oligosaccharide of DP <25 [24].

The aim of this study was to demonstrate that inulin-type fructans with a DP >25 could be extracted from different artichoke wastes by employing an ultrasound extraction method including a precipitation step of inulin fibers in ethanol, which makes such wastes easily treated during manufacturing processes of prebiotic and supplements [25]. Likewise, the main goal of the study was to evaluate that the isolated long chain inulin-type fructans extracted from artichoke waste could sustain the growth of selected strains belonging to different* Lactobacillus* and *Bifidobacterium *species, previously characterized as probiotics [26].

## 2. Materials and Methods

### 2.1. The Powder Preparation of Artichoke Wastes

Artichoke materials (globe artichoke *Cynara scolymus*) were obtained from local market and from the company Flanat Research Italia Srl. Artichoke materials were cleaned with distilled water, chopped, and finely ground in a blender. They were then dried in oven at 70°C overnight to constant weight, crushed, and stored at 4°C until use.

### 2.2. Extraction of Artichoke Inulin

The process of inulin-type fructans extraction from artichoke bracts, stems, leaves, and heart (as control) by ultrasound treatment was performed according to the protocols described by Lingyun et al. [27] and by Moerman et al. [28], with some modifications, using a Digital ultrasonic bath Mod. DU-65 (Agro Lab, Italy). In details, 3.3 g of dried powder were dispersed with 10 mL of distilled water in a 50 mL falcon tube. The sample tube was immersed in an ultrasound cleaning bath (Hz 40) and the liquid level inside the tube was about 1.0 cm below the liquid surface in the bath. The extraction was performed for 40 min at 70°C and the extracted slurry was filtered with a Buchner funnel using filter of 43–48 *µ*m to collect the supernatant. Precipitation with ethanol (≥99%, Sigma) was carried out to isolate inulin-type fructans present in the sample. After addition of 2 volumes of ethanol, the supernatant was stored at −20°C overnight. The precipitate was collected by centrifugation at 6000x g for 15 min and then oven-dried at 50°C to eliminate the ethanol. Finally, the sample was resuspended in 2 mL of distilled water and freeze-dried. The freeze-dried samples were kept stored at 4°C until use.

This method was compared with other two methods at laboratory scale to simplify the first step of the extraction method. The two methods consisted of: (1) a maceration step for 2 h at 70°C; (2) a maceration step at room temperature for 2 h after heating at 70°C the sample for 10 min. Then, in both the extraction methods the procedure concerning the second step was performed as described above.

### 2.3. Determination of Inulin-Type Fructans Content

The quantification of fructans in the freeze-dried material was assessed by the fructan assay procedure kit Megazyme [29]. The procedure was performed according to the manufacturer instructions. The inulin-type fructans concentration was calculated taking into account the fructose, glucose, and sucrose contents in the artichoke extracts before and after hydrolysis with fructanase. The samples were treated with a specific sucrase/maltase enzyme to completely hydrolyze saccharides to D-glucose and D-fructose. The reference values of samples were determined by direct analysis of D-glucose plus D-fructose using the hexokinase/phosphoglucose isomerase/glucose 6-phosphate dehydrogenase analytical procedure. The amount of NADPH formed in this reaction is stoichiometric with the amount of D-glucose plus D-fructose. NADPH formation is measured by the increase in absorbance at 340 nm. The fructan content of samples was determined after hydrolyzation to D-fructose and D-glucose by endo- and exo-inulinases, and then D-fructose and D-glucose content was measured as described above. Therefore fructan content was determined by subtracting absorbance values of the reference from those of the sample. Before each enzymatic assay samples were heated for 30 min at 50°C to ensure sample complete dissolution. Data are reported as means (±SD) of three measures for each sample.

### 2.4. Determination of Phenolic Compounds Content

Total phenolic content of the extracts was estimated using Folin–Ciocalteu phenol assay previously described [30]. Standard solutions (0–100 *µ*g/mL) of Gallic Acid (GA) were used for the calibration. The GA solutions were prepared in 80% methanol (Sigma), and the absorbance values were measured at 765 nm. For sample measurement, 0.5 mL (1/10 dilution) of Folin–Ciocalteu phenol reagent and 1 mL of distilled water were added to 100 *μ*L of artichoke extract. The solutions were mixed and incubated at room temperature for 1 min. Then, 1.5 mL of 20% sodium carbonate (Na_2_CO_3_) solution were added to the sample, mixed and after incubation for 120 min, absorbance was read at 765 nm against blank. Results were expressed as mg of Gallic Acid Equivalent (GAE)/g of freeze-dried extract. Data were reported as means (±SD) of three measures for each sample.

### 2.5. Determination of Proteins Content

Protein content of the artichoke extract was determined according to the dye binding method of Bradford. A calibration curve using bovine serum albumin as standard was performed to determine the protein concentration of the extracts.

### 2.6. Determination of the Degree of Polymerization (DP_n_)

Artichoke extract sample was prepared at a concentration of 1 mg/mL in deionized water. The degree of polymerization (DP) of fructans in the sample was analyzed by Agilent 1100 HPLC equipped with a Agilent Refraction Index Detector. A PolySep-GFC-P 5000 Phenomenex column (7.8 mm × 300 mm) was used with ultrapure degassed water as the mobile phase at a flow rate of 0.8 mL/min. The column temperature was set to 25°C and injection volume was 20 *µ*L. Thus, 4 polysaccharides standards, 3 dextrans, and 1 nystose, were chosen for their specific molecular weights and for their similarity to the inulin molecular structure.

Dextran standards were prepared at the same concentration of the artichoke sample. The artichoke sample and the standards were analyzed at the same chromatographic conditions. Finally, a standard curve was determined with the retention time (RT) of Dextran as the abscissa, and the logarithms of molecular weight (log MW) as the ordinate. The DP of inulin-type fructans in the artichoke samples was calculated from the corresponding MW.

### 2.7. Bacterial Strains, Media and Substrates

The bacterial strains used in this study are cited in Table 1. The strains were selected for their established nature of probiotics and for their probiotic properties [26]. They were provided from a private collection of the company Roelmi Hpc. Probiotic bacteria were activated by growing it in MRS broth supplemented with 0.05% L-cystein for 48 h under anaerobic conditions using Anaerocult A GasPac system (Merck, Darmstadt, Germany) at 37°C. Microbial cells were then transferred at 1% inoculation concentration (v/v) in MRS broth medium followed by incubation for 24 h prior to use. Modified MRS medium, without glucose and supplemented with 0.5 g/l L-cysteine hydrochloride (Conda), hereafter referred to as mMRS medium, was used as the basal fermentation medium throughout this study. The pH of the medium was adjusted to 6.8 before sterilization (121°C for 15 min). Glucose (Sigma), fructooligosaccharide FOS (DP ~ 3–5) or artichoke extract was added to mMRS as the sole carbon source (2%, wt/vol). In all cases, these sugars were sterilized through membrane filtration using Millex® Syringe Filter Units (pore size, 0.45 *μ*m; Merck Millipore, Darmstadt, Germany) and added aseptically to the sterile mMRS medium.

### 2.8. Growth Experiments for Prebiotic Effect with Lactobacillus and Bifidobacterium Strains

All probiotic bacteria cited in Table 1 were inoculated into MRS Agar 48 h prior to use. Glucose, fructooligosaccaride (FOS DP 3–5) or artichoke extract were added individually before inoculation to give a final concentration of 2% wt/vol. Sterile falcons containing 10 mL of modified MRS broth medium were inoculated with 100 *μ*L of a solution of each bacterial strain (O.D. 0.1), mixed, capped and introduced into anaerobic jars. The anaerobic jars were incubated at 37°C. Samples were removed at 48 h to measure the OD_600_.

### 2.9. Statistical Analysis

Experiments were performed in triplicate and results were presented as mean values ± standard deviation. The statistical relevance was assessed by Student's *t* test. The significance was defined as ^∗∗^*p* value <0.05 and ^∗^*p* value <0.1.

## 3. Results

### 3.1. Selection of the Artichoke Wastes

The artichoke industry generates a solid waste consisting mainly of stems and external bracts of the flowers, which are about the 70% of the total flower, and the leaves of the plant, which are mostly discarded. Initially, we decided to analyze all the three artichoke wastes as potential raw materials for the successive analyses. The three artichoke parts were extracted by an ultrasound-assisted method in aqueous solution at 70°C. In these first experiments, we observed a significant content of extracted inulin-type fructans (determined as described in materials and methods) only from two parts of the waste, external bracts, and stems (Table 2). This preliminary extraction was also performed on the heart of the artichoke as positive control, because it is known that this part of the artichoke is the most rich in inulin-type fructans.

### 3.2. Extraction of Inulin-Type Fructans from Artichoke

On the basis of the preliminary results, the artichoke wastes utilized for the inulin-type fructans extraction were the bracts and the stems.

To enhance the yield of long chain inulin-type fructans, we decided to employ a method to obtain a higher amount of these compounds from bracts and stems, with particular attention towards the precipitation step that could have an effect on the length of the inulin chain. For this purpose, we added to the ultrasound-assisted method in aqueous solution at 70°C, a precipitation step with EtOH, 2 : 1 ratio of each extracted sample. After centrifugation and lyophylization, we measured the amount of dried weight of each sample from each artichoke part. Then, each sample was subjected to the enzymatic assay with fructanase, in order to indirectly determine the amount of inulin-type fructans present in the extracts. Moreover, the extracts were also analyzed for their content of polyphenols, and proteins. The content in inulin-type fructans, free reducing sugars, polyphenols, and proteins in the two parts of artichoke is listed in Table 3. It shows that a similar inulin-type fructans composition was obtained from both the artichoke stems and bracts. Inulin-type fructans concentration was over the 70% on dry precipitate, total reducing sugars ranged near 5%, while phenolic compounds and proteins constitute 5-6% and 4%, respectively. These results indicate that waste parts of artichoke have a high potential for further development as commercial prebiotics, due to their high content of indigestible polysaccharides.

### 3.3. Properties of Artichoke Extracts

**Figure 1:**
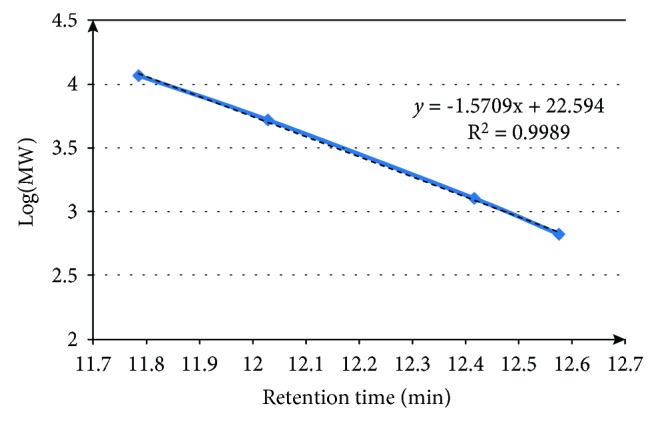
Calibration curve of molecular weight of the polysaccharides standards, 1: Nystose (666 Da), 2: Dextran (1270 Da), 3: Dextran (5220 Da), 4: Dextran (11600 Da).

**Figure 2:**
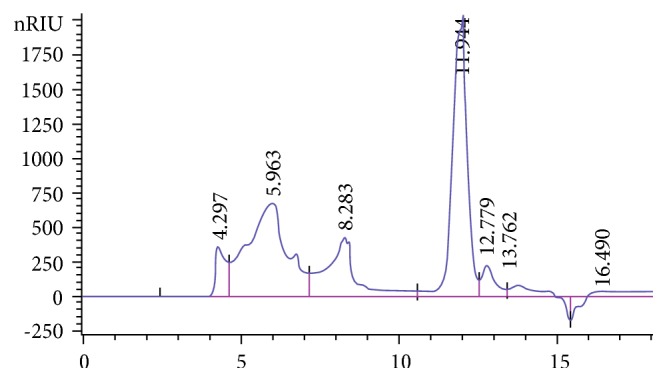
HPLC-RID chromatogram of inulin from globe artichoke waste extract (stem).

Extracts of bract and stem samples obtained with the extraction conditions previously described, were analyzed by SEC-HPLC to obtain the MW of the inulin-type fructans. For this, a calibration curve of 4 standards of known molecular weights was prepared as described in Materials and Methods. The elution profile of the standards was used to determine a calibration curve, correlating the retention time to the Log MW of each standard; the coefficient of linearity was determined and the equation: *y* = −1.5709*x* + 22.594; *R*^2^ = 0.9989, indicated a good correspondence between the two variables (Figure 1). The MW of the inulin-type fructans of the bract and stem waste extracts was determined by establishing the retention time of the peaks in the chromatogram and then calculating the MW using the above equation. Since the inulin structure consists of a linear backbone of repetitive fructose moieties linked to one terminal glucose moiety, the DP was determined by dividing the MW of the inulin polymer by the MW of fructose monomer. The retention time distribution of the extract of bract and stem samples is quite similar. As example, we report the profile of the stem in the Figure 2, where the graph shows a principal peak with DP average of 32–42 which represents the inulin; the other peaks show retention times which do not correspond to the uttermost DP of inulin polymers occurring in globe artichoke.

### 3.4. Prebiotic Effect of Artichoke Inulin-Type Fructans

**Figure 3:**
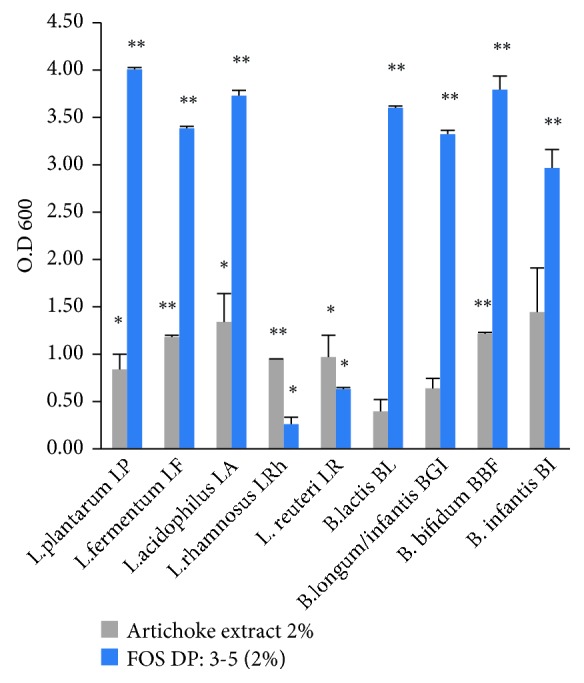
The OD_600_ of *Lactobacillus* and *Bifidobacterium* strains cultures after 48 h incubation at 37°C under anaerobic condition in the presence of 2% artichoke inulin (grey) and FOS (blue). Data represent the mean of three independent experiments, each performed in duplicate. Error bars indicate the mean standard deviation for each culture. ^∗∗^*p* value <0.05 and ^∗^*p* value <0.1.

In order to prove that artichoke inulin-type fructans are effectively fermented by *Bifidobacterium and Lactobacillus* strains originally isolated from the human colon, we have studied the effect of their addition to cultures containing selected strains of bifidobacteria or lactobacilli (Table 1), starting with an OD_600_ of 0.1 at the beginning of the experiment and by measuring the OD_600_ of the cultures after 48 h of anaerobic fermentation (Figure 3). Similar cultures with commercial fructooligosaccharides (FOS) of DP 3–5 were considered as a control. Figure 3 shows that all the tested strains were able to ferment high molecular weight inulin of DP 32–42 and short chain FOS (DP 3–5). For all the strains except *L. rhamnosus* and *L. reuteri* the highest OD was obtained when FOS was used as the growth substrate. Indeed, these two strains grew to a smaller extent on FOS comparing to the other strains, while apparently their growth on artichoke inulin-type fructans was greater than that on FOS at the end of the incubation. Besides, the five *Lactobacillus* and four *Bifidobacterium* strains fermented inulin-type fructans in a comparable extent. Therefore, these findings show no significant difference among these strains, belonging to two different genera and different species, in the ability to ferment artichoke long chain inulins.

### 3.5. Experiments toward a Method for the Industrialization of the Artichoke Inulin-Type Fructans to Use as Prebiotics

Previous results indicated that artichoke bracts and stems could be considered for biotechnological exploitation as raw materials for the extraction of the artichoke inulin-type fructans to use as potential commercial prebiotics. In order to industrialize the preparation of the extracts, we decided to test two different methods at laboratory scale to simplify the extraction method preserving the content of inulin-type fructans. The two methods consisted of: (1) a maceration step for 2 h at 70°C; (2) a maceration step at room temperature for 2 h after heating at 70°C the sample for 10 min. Then, in both the extraction methods the procedure concerning the second step was performed as described above. The extracts obtained from both bracts and stems with the two methods, were then analyzed with the fructanase-based enzymatic assay, and compared with each other. Results are reported in Table 4. Data show that the content of inulin-type fructans obtained is higher from the stems when we used the same method. Otherwise, comparing the different content of inulin-type fructans obtained with the two methods, we can observe that the first one is more efficient than the second with respect to the yield of inulin-type fructans content from both bracts and stems of the artichoke.

## 4. Discussion

Inulin polymers of plant origin are a subject of interest in many food research programs, for their low food caloric value and their dietary fiber effects. They are a polydisperse *β*-2, 1 fructans with varying degree of polymerization which determines their physicochemical properties and thus their functional characteristics [9, 10]. Besides, it has been demonstrated that these naturally occurring polymers exist as a reserve substance in the bract and stem of artichokes [18–20, 31]; these parts of the flower are very interesting in the artichoke industrial wastes.

In the literature few data regarding artichoke wastes are present, and the most only consider the extraction of the short chain inulin-type fructans [21, 31]. Thus, we would like to demonstrate that inulin-type fructans with a DP > 25 could be extracted from different artichoke wastes exploiting a combined approach of an ultrasound extraction step and a precipitation step of inulin fibers in ethanol, making them easily treated during the extraction processes of prebiotics.

Our approach produced an extract from the different parts of the artichoke rich at the 70% of inulin-type fructans with a DP > 10 (DP between 32 and 42). In our knowledge, only Machado et al. [21], using ultrasound indirect and direct sonication extraction to recover inulin from globe artichoke bracts waste, reported a yield of more than 50%. Besides, the degree of polymerization of inulin obtained in our study is much higher than that reported by the aforementioned study. This result is also consistent with the report by Moerman et al. [28], who demonstrated that by precipitation with ethanol, the DP of recovered compounds could be raised from 8.1 to 25 for chicory inulin and from 29 to up to 40 for dahlia inulin. Moreover, Terkmane et al. [32] showed that ethanol affects the solubility of inulin by affecting its shape and size in the extraction medium in favor of high molecular weight inulin from globe artichoke.

Therefore, the artichoke waste extracts obtained by this method, produced inulin-type fructans with a DP value of 32–42 which is comparable to available commercial inulin from chicory source (DP = 36) which make their properties similar. Moreover, the same acceptable yield of inulin-type fructans could be obtained from both bracts and stems of the artichoke wastes.

The prebiotic effect of inulin has been well proven [9, 33]. It lies in stimulating the growth of beneficial bacteria in the intestine. Among those, bifidobacteria and lactobacilli are the most known. Moreover, the inulin fermentation by those bacteria generates metabolites with health benefits to the host [14]. In order to demonstrate the prebiotic effect of our artichoke extract, the ability of several microbial strains belonging to* Bifidobacterium *and* Lactobacillus *genera to grow on artichoke inulin has been investigated *in vitro*. At the same time, as positive control, we evaluated the ability to grow also on commercial FOS. All tested strains were able to grow on artichoke inulin and commercial FOS (Figure 3). However, of the five tested *Lactobacillus* species, *Lactobacillus plantarum,Lactobacillus fermentum,* and *Lactobacillus acidophilus* grew well on FOS, whereas *Lactobacillus reuteri* and *Lactobacillus rhamnosus* grew to a smaller extent; while the level of growth on artichoke long chain inulin of all the strains was comparable. These results suggest that all these strains present metabolic pathways of degrading higher DP inulin and FOS.

*Lactobacillus* strains have been reported to possess two alternative pathways for the metabolism of sucrose and higher FOS through intracellular hydrolysis [34]. In contrast, an extracellular hydrolysis of sucrose, oligo, and polysaccharide is less frequently found in this genus. For instance, among several *Lactobacillus* strains, only *Lactobacillus reuteri* SD2112 presented extracellular enzymes able to degrade sucrose, FOS, and polysaccharides as reported by Ganzle and colleagues [34].

The degradation of inulin by extracellular enzymes is not the only reported mechanism. For instance, *L. delbrueckii* TU-1 and *L. delbrueckii* JCM 1002 hydrolyze inulin without possessing an extracellular *β*-fructosidase [35]. Since these two strains fail to degrade fructose, the authors suggested the presence of a transporting system in which larger molecules of inulin are more preferably taken up into the cells where their hydrolysis occurs.

In our fermentation experiments, *Lactobacillus* strains show a similar growth behavior toward inulin and FOS with respect to the literature mentioned above, suggesting the presence of different sugars metabolic mechanisms.

The degradation of artichoke inulin by the four tested *Bifidobacterium* strains was comparable, although their growth on FOS substrate was much higher. Unlike lactobacilli, in which sugar metabolism is mostly restricted to intracellular enzymes, bifidobacteria have been reported to maintain a more extensive tool set for extracellular hydrolysis and transport of complex carbohydrates sugars [36, 37]. Our growth results of the four *Bifidobacterium *strains on FOS and inulin are in accordance with previous reports, where it was demonstrated that the majority of bifidobacteria are able to degrade FOS and inulin by extracellular fructosidases, whose induction is dependent on the type of sugar used as growth substrate [37]. For instance, *Bifidobacterium adolescentis* ALB 1 activated extracellular fructosidases when grown on inulin rather than FOS [22].

All our results highlighted for the first time that long chain inulin-type fructans can be extracted from artichoke wastes with a satisfying yield and that they can be fermented by different probiotic species, thus they could be used as prebiotics.

**Figure 4:**
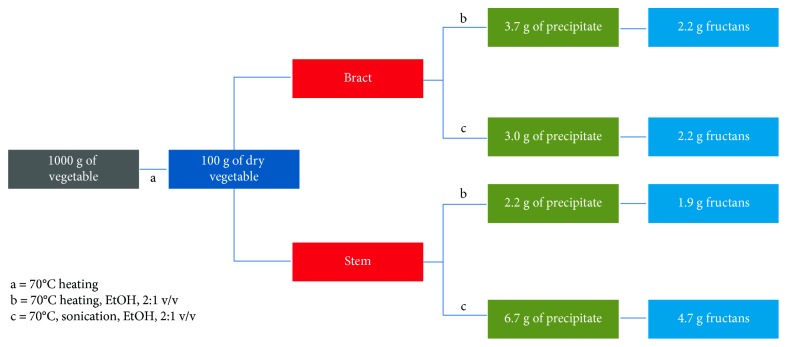
Flow chart of the inulin content (g) in bract and stem parts obtained from 1 kg of fresh artichoke waste using different methods. (blue box) Dried artichoke residue obtained by heating fresh artichoke at 70°C; (green box) inulin content obtained by precipitation with EtOH, 2 : 1 v/v; (light blue box) fructans content measured in precipitated inulin. (a) are the conditions used for the water elimination from the plant parts; (b) and (c) are the two acceptable conditions used to recover the high DP inulin from the dried parts (see text).

In this perspective, we developed a process of the inulin-type fructans extraction from artichoke wastes, in order to obtain a simpler method to industrialize the production of inulins. We performed the extraction of artichoke inulin with two different methods (Table 4), showing that only the maceration step heating at 70°C was enough efficient. From here, considering the ultrasound and the maceration methods as alternatives, we can add some considerations. A summary of the yield of extraction with the most acceptable methods is reported in the Figure 4. Looking at Figure 4 we can note that there are differences both in the amount of retrieved mass from different origins and in the percentage of recovered inulin-type fructans. From bracts the two alternative methods give very similar results, nearly the same dry weight and exactly the same amount of fructans. In contrast, from the stems the sonication method is much more efficient (two times more dry extract and one and half more fructans). The conclusion is that both the alternative methods provide the recovering of a inulin-type fructans enriched fraction from both wastes; although, sonication is the method of choice to get the best result. However, from an industrial perspective, the maceration of large amount of waste is more feasible than sonication.

In conclusion, this study represents a first step to develop a simpler method for the production of long chain inulin-type fructans from the artichoke wastes and to prepare synbiotic formulations by combining artichoke inulins with different *Lactobacillus* and *Bifidobacterium* species for further studies, including *in vitro* gut simulation models and *in vivo* trials.

## Figures and Tables

**Table 1:** Bacterial strains used in this study.

Strain	Source
*Lactobacillus acidophilus* LMG P-29512 (formerly DSM 24936)	Human
*Lactobacillus fermentum* DSM 25176	Human
*Lactobacillus reuteri* DSM 25175	Human
*Lactobacillus plantarum* DSM 24937	Human
*Lactobacillus rhamnosus* LMG P-29513 (formerly DSM 25568)	Human
*Bifidobacterium animalis* subsp. *lactis* LMG P-29510 (formerly DSM 25566)	Human
*Bifidobacterium longum* subsp. *longum* DSM 25174	Human
*Bifidobacterium longum* subsp. *infantis *LMG P-29639	Human
*Bifidobacterium bifidum *LMG P-29508	Human

**Table 2:** Sugar composition of the dried artichoke waste residue.

Artichoke parts	Reducing sugars content (g/100 g of dry material)	Inulin content (g/100 g of dry material)
Bract	10.40 ± 0.91	15.96 ± 1.75
Stem	20.68 ± 2.30	27.97 ± 4.58
Leaves	23.09 ± 0.84	4.70 ± 1.21
Heart	30.59 ± 2.02	40.02 ± 0.99

**Table 3:** Chemical composition of the artichoke precipitated waste residue isolated from bracts and stems^a^.

Artichoke parts	Reducing sugars content (g/100 g of dry material)	Inulin content (g/100 g of dry material)	Phenolic compounds (g/100 g of dry material)	Proteins (g/100 g of dry material)
Bract	5.66 ± 0.99	70 ± 6.3	6.3 ± 0.2	4.05 ± 0.09
Stem	5.44 ± 0.81	70 ± 1.2	5.1 ± 0.3	4.1 ± 0.09

^a^The weights are referred to 100 g of precipitate.

**Table 4:** Sugar composition of the artichoke precipitated waste residue prepared with the two different methods described in the text^a^. 1 and 2 are the two methods as follows: (1) a maceration step for 2 h at 70°C; (2) a maceration step at room temperature for 2 h after heating the sample at 70°C the sample for 10'. For both the methods the samples were filtered and submitted to precipitation with 2 volume of ethanol and after centrifugation were dried in oven at 70°C.

Artichoke parts	Reducing sugars content (g/100 g of dry material)	Inulin content (g/100 g of dry material)
Bract.1	4.369 ± 0.024	58.26 ± 5.46
Stem.1	6.161 ± 0.363	85.87 ± 0.19
Bract.2	5.469 ± 0.086	27.46 ± 3.79
Stem.2	6.285 ± 0.083	54.16 ± 1.98

^a^The weights are referred to 100 g of precipitate.

## Data Availability

The experimental data used to support the findings of this study are included within the article.
